# Measuring visual exposure to smoking behaviours: a viewshed analysis of smoking at outdoor bars and cafés across a capital city’s downtown area

**DOI:** 10.1186/1471-2458-14-300

**Published:** 2014-04-06

**Authors:** Amber L Pearson, Daniel Nutsford, George Thomson

**Affiliations:** 1 Department of Public Health, University of Otago, PO Box 7343, 23A Mein Street, Newtown, Wellington 6242, New Zealand; 2 Department of Geography, GeoHealth Laboratory, University of Canterbury, Private Bag 4800, Christchurch 80140, New Zealand

## Abstract

**Background:**

The influence of visual exposure to health-related behaviours, such as smoking, is increasingly acknowledged in the public health literature. Social contagion or normalisation is thought to operate through the visibility of those behaviours. There has been a lack of systematic and comprehensive approaches to quantifying visual exposure to these behaviours over a relatively large geographic area. We describe the novel application of a geographic tool, viewshed analysis, to estimate visual exposure to smoking outside bars/cafés across a downtown area.

**Methods:**

Smoking was observed for different times and days of the week at 14 outdoor areas of bars/cafés throughout downtown Wellington, New Zealand. We used these data to extrapolate to other bars/cafés with outdoor seating. We then conducted viewshed analyses to estimate visual exposure to smoking at bars/cafés for all public outdoor spaces.

**Results:**

We observed a smoking point prevalence of 16%. Visibility analyses indicated that estimated visible smoking was highest in the evenings (7-8 pm), where the average values across Wednesday and Friday ranged from zero up to 92 visible smokers (mean = 1.44). Estimated visible smoking at midday ranged from zero to 13 (mean = 0.27). Values were also higher at the end of the week compared with midweek in the evening. Maps indicate that streets with high levels of retail shops and hospitality areas had high values of estimated visible smokers, particularly in the evening where numbers were consistently above 50.

**Conclusions:**

This paper highlights a useful method for measuring the extent of visual exposure to smoking behaviours across relatively large areas using a geospatial approach. Applying this method in other locations would require consideration of place-specific characteristics which impact on visibility and could be improved through more sophisticated extrapolation of observational data across the study area. The findings of this and similar research could ultimately support the expansion of smokefree public spaces.

## Background

There is emerging evidence of the influence of visual exposure to harmful or helpful health-related behaviours, such as smoking or engagement in physical activity, in the public health literature. These studies tend to focus on the social contagion or normalisation of behaviours through their observed presence in particular places. For example, studies on physical activity indicate that observing others engaging in physical activity in one’s neighbourhood was associated with increased walking and a lack of observing others engaging in physical activity was associated with a negative perception of exercise for individuals [1]. Likewise, research on observed smoking indicates an association between the frequency of observed smoking in some locations and the perception that smoking is socially acceptable, particularly for youth [2,3]. The normality of smoking can reduce the likelihood of quit attempts and successful quitting [4-6]. Social ambience, including smoking visibility, may influence smoking relapse, particularly at bars and cafés [7]. Policies aimed at smoking prevalence reduction increasingly involve the de-normalisation of smoking via smokefree policies in public outdoor places [8].

While there has been a range of studies that have measured the extent of smoking outdoors for particular types of venues such as parks or bus stops [9], there is a lack of research presenting a systematic and comprehensive spatial approach to quantifying visual exposure to health-related behaviours over a relatively large geographic area. Over the past 15–20 years, advances in geographic techniques for automating the measurement and display of viewsheds (visible areas) have facilitated their use in landscape ecology [10], archeology [11], and site selection for minimal visual impact [12]. In viewshed analyses, one or more observation points are used to determine which cells in an input raster (a digital matrix of cells or pixels) can be seen from each location. All cells are assumed visible unless there is an intervening feature between itself and the observer point. Typically, viewshed analyses rely on topography for determining visibility of various locations (e.g., from a hilltop). However, in small scale urban settings, particularly those that are relatively flat, buildings may be more important obstructions to visibility. In some research observational or photographic data are combined with elevation or visual obstruction data to quantify and represent visibility of objects in viewshed analyses. In the case of smoking behaviours, quantification of visibility allows for measurement at various locations throughout the study area and for highlighting differential exposure, including hotspots of harmful visual exposure. These spatial data can be used in tandem with data, say, on distributions of populations (whether resident or frequent visitors), whose health could be influenced or harmed by the behaviour.

As there has been a lack of mapping of outdoor smoking or its visibility over large areas, we aimed to use viewshed analysis to understand and quantify visual exposure to this harmful behaviour. This quantification is useful for highlighting areas of high exposure and may aid in expansion of smokefree public spaces, as reductions in outdoor visibility of smoking may reduce cues to smoke.

## Methods

### Project area

**Figure 1:**
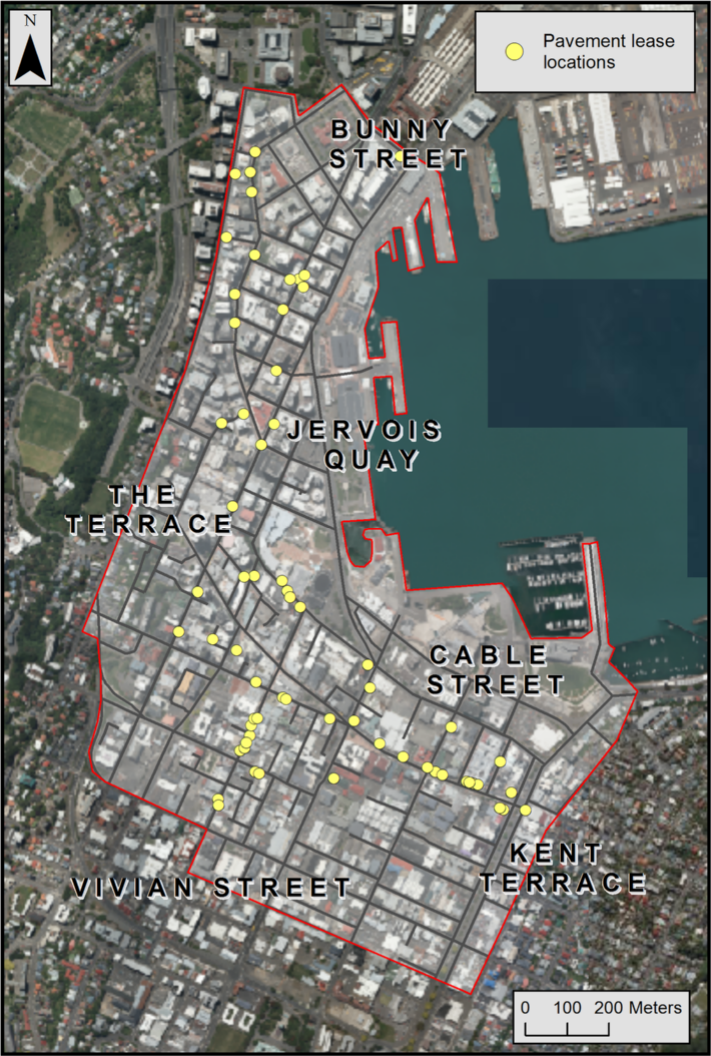
Study area: Wellington central business district and its bounding streets, with all pavement lease locations (n = 70).

The study site was the central business district (CBD) of Wellington City, New Zealand, demarcated by street perimeters (Figure 1). The CBD is an area of high volumes of pedestrian traffic, shopping, nightlife, dining and recreation due to its compact urban design. The relatively flat topography, narrow streets and limited parking mean that foot traffic is the most common source of transportation and this increases the potential for visual exposure to smoking in the downtown area. In Wellington smoking is currently permitted in outdoor public spaces (except for parks and playgrounds) and in the outdoor dining areas at bars/cafés.

### Data included in the viewshed database

Visibility or viewshed analysis is a method for quantifying visible areas across a geographical region. By providing a computer program with a representation of terrain and building footprints and heights, one is able to determine which areas are visible from defined observer locations. Typically, a Digital Elevation Model (DEM) is included in this type of visibility analysis to represent terrain, however, nearly all the Wellington CBD is located on flat terrain, nestled between the coastline of Wellington Harbour and low lying hills. As such, incorporating a DEM was not necessary for the viewshed analysis in this study.

Rather, using a computer program, we generated a digital grid of 1 metre by 1 metre cells or pixels (called a raster file) across the study area to represent ground level elevation. This gridded raster file provides the base layer for visibility analysis in the program. Each cell/pixel is treated as an independent entity which may or may not be visible from defined observer locations. Then, various visibility obstructions were added to this digital layer. Extruded building footprints obtained from the Wellington City Council (2012) were merged with the raster file to represent the CBD in a 3-dimensional space.

Next, we added a geographic data layer of the locations of outdoor smoking. We compiled all hospitality business addresses with pavement licences registered with the City Council. These addresses within the CBD were geocoded (n = 70) using ArcGIS v10 (Redlands, CA, USA). These pavement license points were located on the ground level raster file and then were assigned an elevation of 1.5 metres (i.e., average height of a seated patron) for the 3-dimensional viewshed analysis. The locations of streets with high numbers of retail shops and hospitality venues were digitally added to the base map, based on local knowledge of the area.

We then defined observer locations (viewpoints) as all locations within public areas (e.g., sidewalks along streets, parking lots, streets, parks, etc.). These data allowed us to calculate the number of visible smokers (occurring at outdoor bar/dining areas) for each observer location, accounting for obstructions in vision caused by the presence of buildings.

### Observational data collection

Observational data were collected in order to estimate the prevalence of smoking in licensed pavement areas of bars/cafés/restaurants [13]. Different observational times were used to capture temporal fluctuations, both by time of day and day of week. Methods for observations followed methods trialled in a previous study [9], but were slightly modified to increase data accuracy. Data were collected over 15-minute periods twice per day (during 12-1 pm and 7-8 pm) on Wednesdays and Fridays over two consecutive weeks in April 2013, for a total of eight observation periods for each location. Sites were selected for observation from the list of licenced pavement leases within the CBD. Selected sites operated between the hours of 11 am and 11 pm. Nineteen sites met the inclusion criteria, of which 14 were randomly selected (using a random number generator using Excel 2010 software). Fourteen was the maximum number of sites that could be observed with the resources available, as two sites each were assigned to seven pairs of observers. At each observation period, the number of patrons and number of lit cigarettes at five minute intervals were recorded, using 30-second scans. To minimise conspicuousness, counts of smokers were tallied using smartphones with note-taking applications such as ‘Note’ and ‘S Memo’. From these observations, averages, and ranges were calculated by time of day and day of the week for the 15-minute observation periods [13].

### Extrapolation from observed sites to other sites

In order to assign smoking levels to venues for which we did not collect observational data, we divided the pavement licenses into three categories: Bars, Cafés and Restaurants. Then, using the observational data, we calculated averages for each category by daytime, night time, mid-week and end of week. We then assigned these averages to the remaining venues according to their category.

### Viewshed analyses

Visibility analyses were conducted to provide a smoking visibility index for the Wellington CBD using the software ArcGIS v10 (Redlands, CA, USA). The purpose of the viewshed tool is to classify a region into visible or non-visible areas from a single or multiple defined observer points. This is achieved by generating lines of sight (LoS) between observer point(s) to pavement lease locations, accounting for obstructions using the gridded raster surface. Typically, viewshed analysis produces a binary raster file (1 for visible cells, 0 for non-visible cells). However, in order to reflect the number of smokers visible, positive cell values were multiplied by the number of smokers for a specific pavement lease based on the observational data.

A novel iterative script (see supplementary material) was developed in Python programming language to conduct the viewshed analysis outlined here. The output raster maps highlighting which areas were visibly exposed to smoking from each pavement license were consecutively overlaid, resulting in maps of the cumulative exposure to visible smoking across the CBD. In order to identify weekly and diurnal variation in smoking visibility, the process was independently repeated six times, to estimate visible smoking based on observed smoking counts during four different time periods. This method produced an estimated number of visible smokers (located at pavement bars/dining areas) from the viewpoint of each defined observer location – in public areas (roads, sidewalks, and other public spaces) throughout the CBD in a 15-minute period. Specifically, the following maps and estimated visual exposure to smoking at pavement licenses *from a given viewpoint* were generated:

a) Visibility of smoking at pavement licenses at midday (12-1 pm - Average of Wednesday and Friday)

b) Visibility of smoking at pavement licenses in the evening (7-8 pm - Average of Wednesday and Friday)

c) Visibility of smoking at pavement licenses Wednesday or Friday at midday (12-1 pm)

d) Visibility of smoking at pavement licenses Wednesday or Friday evening (7-8 pm)

### Ethics approval

Ethical approval for this research was obtained through the ethics approval process of the University of Otago, in April 2013.

## Results

Over the two-week observation period, a total of 411 smokers were recorded during a total of 28 hours observation time at 14 different pavement leases of bars/cafés/restaurants in the Wellington CBD. The observations were in a range of weather conditions, from rain and wind to warm sunshine. We observed a smoking point prevalence of 16%, with a maximum number of 44 lit cigarettes observed at one venue in one 15-minute observation period and 14 lit cigarettes in a 30-second scan. In the evenings, bars experienced the highest number of observed smokers, with an average of 10 lit cigarettes every 15 minutes, while restaurants and cafés had an average of three. At midday, all three venue types had similar levels of observed smoking. However, levels were all considerably lower than during evening observations, with an overall average of one cigarette per 15-minute period.

**Figure 2:**
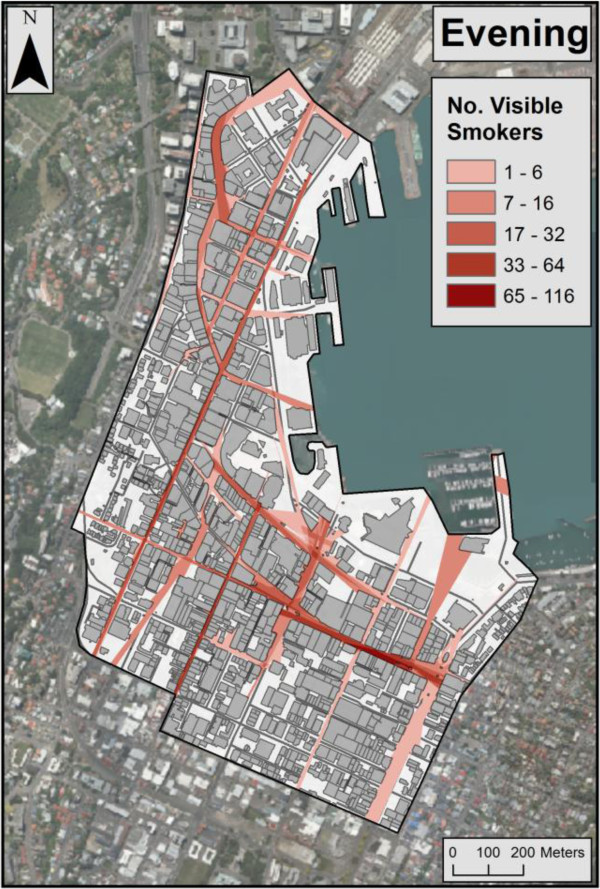
Average estimated visibility (from all public areas) of smoking occurring at hospitality pavement leases in Wellington CBD 7-8 pm (average of Wednesday and Friday estimates).

**Figure 3:**
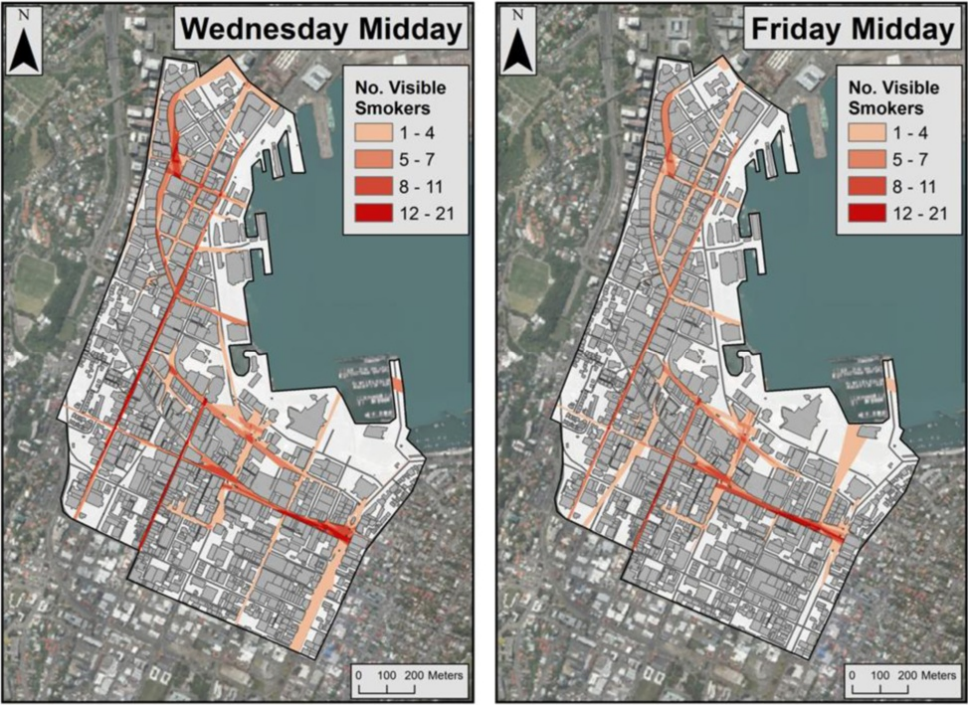
A comparison between Wednesday and Friday midday (12-1 pm) estimated visibility (from all public areas) of smoking occurring at hospitality pavement leases in Wellington CBD.

**Figure 4:**
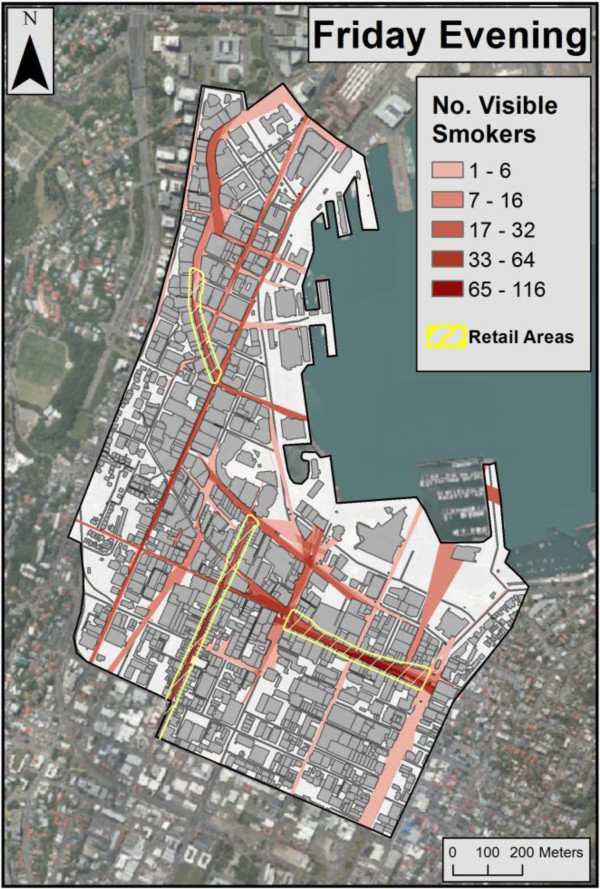
Estimated visibility (from all public areas) of smoking occurring at hospitality pavement leases in Wellington CBD and areas of high retail outlets, Friday (7-8 pm) (mean value = 35 within the yellow areas).

Visibility analyses indicated that estimated visible smoking occurring at pavement leases was highest in the evenings (7-8 pm), where values ranged from zero up to 92 visible smokers (for a particular viewpoint) when averaging Wednesday and Friday evening estimates (average = 1.44) (Table 1). In contrast, estimated visible smoking at midday ranged from zero to 13 across the CBD public spaces (average = 0.27). Figure 2 shows the high levels of estimated visual exposure on evenings (max = an average 92 smokers across Wednesday and Friday). Estimated visible smoking values were also higher in the evening at the end of the week compared with the middle of the week. Figure 3 indicates that estimates for Wednesday midday include larger concentrated areas of higher smoking visibility values than for Friday, particularly in the northern quadrant. This is also reflected by the higher average on Wednesday midday for visible smoking across the CBD (Table 1). Maps also indicate that streets with high levels of retail shops and hospitality venues had high values of estimated visible smokers, particularly in the evening where numbers were consistently above 50. Specifically, the hospitality and retail areas marked in Figure 4 had an average smoking visibility value of 35 across all observation periods.

**Table 1:** Estimated number of visible smokers at outdoor pavement leases in Wellington CBD, by time of day or day of the week

**Time of day or week**	**Estimated visible smoking numbers, for any given public place (1 metre x 1 metre units) in Wellington CBD**		
	***Range***	***Median for all cells with smokers visible***	***Average***
Wednesday midday	0 – 21	3	0.46
Wednesday evening	0 – 76	8	1.26
Friday midday	0 – 20	2	0.34
Friday evening	0 – 116	13	1.70
Midday	0 – 13	2	0.27
Evening	0 – 92	10	1.44

## Discussion

We found the highest estimated visible smoking levels in the evenings, where values reached as high as 92 visible smokers (averaged across Wednesday and Friday). Values were also higher at the end of the week compared with midweek in the evening. During 12-1 pm, when children are more likely to be downtown in public outdoor spaces, our estimated visible smoking levels ranged from zero to 13. Given New Zealand’s smoking prevalence of about 19% [14], countries with higher smoking prevalences, including the UK, the USA and parts of Europe [15], may find that levels of exposure to visible smoking may be even higher. In addition, in places with strong pedestrian cultures, such as parts of Europe, the potential of pedestrians to be visually exposed could be elevated. In addition to the utility of this method to understand smoking behaviours, this method could be applied to measure other health-related behaviours including physical activity or consumption of unhealthy foods.

While almost all large scale visibility analysis scenarios involve the challenge of incorporating complex terrain and vegetation, a host of new problems come to light when studying a built-up environment. Man-made structures, specifically buildings, are difficult to accurately incorporate into terrain models as they create new obstacles and sometimes interesting visual interactions (e.g., reflective glass). However, we included building height and footprint information in our analyses. Dynamic obtrusions such as vehicles and pedestrians also provide a unique challenge, especially in areas of cities with high vehicle and pedestrian volumes which limit the accuracy of visibility analyses. Fortunately, characteristics of the Wellington CBD simplified analyses. We were able to mitigate many of these issues. Firstly, due to the flat terrain of all the streets with pavement leases, we were able to manually generate a high resolution raster to represent the ground surface, which meant terrain had a negligible influence and the process of overlaying accurate building footprints was simplified. Secondly, the heavily built-up nature of the CBD diminished the influence of vegetation which often proves difficult in visibility analyses, due to its varying transparency and height. As such, the application of this methodology in other locations would require consideration of these and other place-specific challenges.

A number of limitations to this research are important to note. Observations were limited to pavement lease areas, and were for only four days in two weeks in April. While this period gave a range of weather conditions, a longer period of observations may provide more representative smoking data. This study did not incorporate smoking at building entrances, or in transit (walking), or that could be seen through windows or on balconies. Studies indicate that entrance areas are important sites of smoking [16]. As such, the visibility of smoking found will be an underestimate of total visible smoking in the CBD. Unrecorded smoking would have occurred with people standing outside hospitality venues without pavement leases, with people walking or standing in other areas of pavement, and in private vehicles. In addition, similar research could develop a more sophisticated method for extrapolation of observational data to locations for which data were not available. For example, land use regression or a similar methodology may produce more reliable, place-specific estimates for smoking based on small area characteristics which promote or hinder outdoor smoking.

A number of assumptions were made to conduct the visibility analyses. Firstly, no distance decay function was used, implying that smokers could be seen regardless of the distance from the viewpoint if a building did not obstruct the view. However, due to the relatively small area of the Wellington CBD (about 1.4 km^2^) and short viewing distances, this was not considered to be a major issue in this study. Still, future analyses could benefit from utilisation of a distance decay function to terminate visibility at a specified distance, to increase realism. Furthermore, analyses were conducted under the assumption that perfect visibility conditions held constant. Visibility can vary under different climatic and diurnal conditions and future research could produce estimates using place-specific climatic data (e.g., the chance of fog, mist and rain) and produce estimates based on realistic night versus daytime visibility. An important strength of this study was the novel application of a geographic tool in quantifying visibility of smoking across a relatively large area.

At present, in Wellington, in other New Zealand cities, and in many other jurisdictions, pavements in downtown shopping areas are not currently smokefree. Those using the pavements are exposed to SHS, and to the normalising of smoking. There are a number of policy options to mitigate this situation. These include smokefree policies for a distance from buildings used by the public (eg., 5 m, which would cover most pavements); smokefree areas near doors and windows of buildings; smokefree spaces where food and/or alcohol is served; smokefree policies for the areas with a number of premises with alcohol and food licenses; and policies for whole smokefree streets or larger areas. Successful examples of policies designated smokefree spaces include legislated requirements for smokefree areas 10 feet from doors, windows and ventilation intakes in all public places and places of employment in Oregon [17], 25 feet in Washington State [18], within 3–4 metres of building entrances in parts of Australia [19-25] and 3–5 metres around workplaces in certain provinces in Canada [26].

The resulting visual data of this method (similar to the Figures presented in this paper) would likely serve as important mobilisers for public policy action and change, particularly at a local level. This is because they provide systematic evidence of visual exposure to smoking at particular places that can otherwise only be conveyed by anecdotal statements or by film/video. These viewshed maps are compelling and can be easily absorbed and interpreted by lay audiences. Health agencies may find this methodological approach of particular interest in conveying a type of health risk to the public and to urban or national policymakers.

## Conclusions

In conclusion, the method highlighted in this research is a useful approach for measuring the extent of visual exposure to smoking behaviours across relatively large areas. Applying this method in other locations would require consideration of place-specific characteristics which impact on visibility and could be improved through more sophisticated extrapolation of observational data across the study area. The findings of this and similar research could ultimately support the expansion of smokefree public spaces.

## Competing interests

The authors do not have any competing interests.

## Authors’ contributions

AP and GT conceptualised the study. GT supervised observational data collection and interpretation. AP supervised the viewshed analyses, conducted by DN. AP drafted the manuscript. All authors edited the manuscript. All authors read and approved the final manuscript.

## Pre-publication history

The pre-publication history for this paper can be accessed here:

http://www.biomedcentral.com/1471-2458/14/300/prepub
